# One-Pot Synthesis of SiO_2_@Ag Mesoporous Nanoparticle Coating for Inhibition of *Escherichia coli* Bacteria on Various Surfaces

**DOI:** 10.3390/nano11020549

**Published:** 2021-02-22

**Authors:** Sukhbayar Gankhuyag, Dong Sik Bae, Kyoung Lee, Seunghyun Lee

**Affiliations:** 1 Department of Electronic Engineering, Kyung Hee University, Yongin city, Gyeonggi-do 17104, Korea; gsukhbayar@khu.ac.kr; 2 Department of Convergence Materials Science and Engineering, Changwon National University, Changwon city, Gyeongsangnam-do 51140, Korea; dsbae@changwon.ac.kr; 3 Department of Bio Health Science, Changwon National University, Changwon city, Gyeongsangnam-do 51140, Korea; kyounglee@changwon.ac.kr

**Keywords:** silver nanoparticles, Gram-negative vs. Gram-positive, antibacterial agents, impregnation method, coating

## Abstract

Silver nanoparticles (Ag NPs) as antibacterial agents are of considerable interest owing to their simplicity, high surface area to volume ratio, and efficient oligodynamic properties. Hence, we investigated the synthesis of silica-supported Ag NPs (SiO_2_@Ag) as an effective antibacterial agent by using a wet-impregnation method. The formation of SiO_2_@Ag with Ag NP (5–15 nm diameter) on the silica particle (100–130 nm diameter) was confirmed with transmission electron microscopy (TEM). The study on antibacterial activity was performed in a liquid culture to determine the minimum inhibitory concentration (MIC) against *Escherichia coli (E. coli)* and *Bacillus subtilis (B. subtilis)* bacteria. Both bacteria are chosen to understand difference in the effect of Ag NPs against Gram-negative *(E. coli)* and Gram-positive *(B. subtilis)* bacteria. SiO_2_@Ag mesoporous nanoparticles had excellent antibacterial activity against *E. coli* bacteria and fully restricted the bacterial growth when the material concentration was increased up to 1.00 mg/mL. In addition, the obtained material had good adhesion to both steel and polyethylene substrates and exhibited a high inhibition effect against *E. coli* bacteria.

## 1. Introduction

Silver nanoparticles (Ag NPs) have received considerable attention owing to their potential for applications such as catalysis, sensing, food packaging, water disinfection, drug delivery, and antimicrobial coatings [1,2,3,4,5,6,7,8,9]. In particular, the biofouling of surfaces in biomedical equipment, wastewater treatment system, and water shipment are adverse effects that result in the growth and aggregation of microbes caused by microorganisms that lead to the extracellular polymer substances (EPS) on the surface [10]. Such an event not only results in an inefficient system but also may cause an outbreak of pathogenic diseases. To overcome this issue, diverse anti-biofouling agents consisting of metal and metal oxide NPs have been used in the past to hinder the accumulation of microorganisms on various surfaces.

Ag NP is an effective material that has been extensively exploited due to its excellent antibacterial properties [11,12]. However, bare Ag NPs are sensitive to environmental factors (e.g., heat, light, and pH) and may exhibit reduced activity over time. In addition, an excessive intake of Ag NPs is known to be harmful to the mammalian organ system [13]. The tendency of NPs to aggregate or oxidize under oxygen-rich environments is also a major drawback. Therefore, there is a considerable need to improve its long-term stability in various environments.

One efficient approach is to load the Ag NPs onto a porous material template to increase their surface area and stability. To maintain the quality of Ag NPs, many researchers used templates (e.g., zeolite, clay, alumina, titania, and silica) to fabricate the silver-containing antibacterial agents [14,15,16,17,18,19]. Among them, silica is a favorable material as a template due to its unique morphology and chemical inertness. Metal NPs are known to attach well to silica, and undesirable agglomeration from external stimuli can also be avoided [20].

While the specific bacteria death mechanism from Ag NP is still unclear, there is a correlation between bacterial cell wall types, the NP size, and its shapes. Zhong et al. prepared stable Ag NPs with different sizes by chemical reduction method and hydrothermal method and observed the highest antibacterial activity with the smallest particle size of 5 nm [21]. Alshareef et al. obtained an excellent antibacterial activity with the truncated and octahedral shapes compared to the spherical Ag NPs which might be due to the higher surface area, active facets, and surface energies [22]. Typically, the bacterial death mechanism could be explained by the penetration of Ag ions into the cell wall which inactivates the DNA replication that leads to bacterial death [23].

Egger et al. [24] prepared silver-silica material-based antibacterial using the industrial flame spray pyrolysis process. The material had an excellent antimicrobial activity for a wide range of bacteria, even though, synthesis route requires an excessive amount of energy to deposit Ag NPs to the silica which makes this approach expensive and complex. Therefore, Matharu et al. [25] achieved a formation of graphene-poly (methyl methacrylate) (GNP) fibers through the preparation of colloidal solution with the combination of gyration process, applied to make the mesh which hinders the bacterial growth. The antibacterial studies have shown that the bactericidal effect increased with higher doses of (8 wt.%) GNP fibers and was effective against E. coli. As for antibacterial usage, environmentally friendly processes and chemicals are preferred to protect the effect of pathogenic disease on the facilities. Hence, a benign, inexpensive, and facile route to synthesize and apply the antibacterial agent is required.

Hybrid nanostructures can also be fabricated through methods such as chemical deposition, layer-by-layer synthesis, reverse micelle, and sol-gel [26,27,28,29,30,31]. Alenezi et al. [32] suggested a new type of approach to loading the metal NPs onto the polymer matrices obtained by pressurized gyration techniques. More recently, Mahalingam et al. [33] created core-sheath configuration through an improved pressurized gyration technique utilizing more than one polymer in a single step on a large-scale technique. However, some of these methods are either expensive, energy-intensive, complicated or require toxic organic substances. Thus, in this work, we suggest a benign and facile impregnation method that utilizes the mechanism of positively charged ions being readily adsorbed to a negatively charged substrate material. Such a method is found to be more simple, less expensive, and requires less toxic reagents [34,35].

Previously, several studies on Ag NPs impregnated on SiO_2_ nanoparticles were reported to understand their effect on antibacterial activity. Kim et al. [36] achieved antibacterial activity applying Ag-SiO_2_ nanoparticles using the Stöber process. However, room temperature synthesis of Ag NPs is nearly impossible with an ammonia base. Moreover, 12 h reaction time is quite long and energy-intensive to obtain homogeneous Ag NPs on the SiO_2_ template and may lead to a high rate of agglomeration that worsens the antibacterial activity. Sujoy et al. [37] presented a significant antibacterial activity with Ag NPs during the process of water purification against planktonic cells. They were able to synthesize Ag NPs after 42 h which is also quite long. Wang et al. [38] noticed that hollow silica nanotubes had better antibacterial activity compared to the nano-silica. The process was also achieved through high-temperature annealing at 600 °C. Such a high-temperature annealing process may lead to higher agglomeration and degradation of mechanical properties. Sotiriou et al. [39] synthesized nanosilver immobilized on nanostructured silica through a flame spray pyrolysis method with a remarkable antibacterial activity. However, some chemicals such as diethylene glucolmonobutyl are quite toxic and the mentioned method is relatively costly and complex.

In this article, we introduce a relatively rapid, benign, and low-temperature one-pot synthesis method to fabricate silica-supported silver nanoparticles (SiO_2_@Ag) with strong antibacterial activity. This material can be applied as a coating medium that inhibits the biofouling effect and bacterial growth. The antibacterial activity of the SiO_2_@Ag mesoporous nanoparticle was investigated by a minimum inhibitory concentration (MIC) test and disk diffusion assay using both steel and polyethylene surfaces.

## 2. Materials and Methods

### 2.1. Materials

Silver nitrate (AgNO_3_, 99.5%), tetraethylorthosilicate (TEOS, 99.8%), trisodium citrate (TSC, 98%). ammonium hydroxide (NH_4_OH, 20–25%), and distilled water (DI H_2_O) were used without further purification. The lysogeny broth (LB) medium containing tryptone, a yeast extract, and sodium chloride (NaCl) were used for the antibacterial activity test.

### 2.2. Synthesis of Silica Spheres

The silica spheres were prepared using the Stöber method [36]. Briefly, 170 mL of ethanol, 0.7 mL of DI H_2_O, 1.1 mL of TEOS, and 7.5 mL of NH_4_OH were mixed and stirred for 4 h at 400 rpm in an ambient atmosphere. Finally, the suspension was centrifuged and dried at 100 °C for 24 h, and ground for further experiments.

### 2.3. Synthesis of SiO_2_@Ag Mesoporous Nanoparticles

**Scheme 1:**
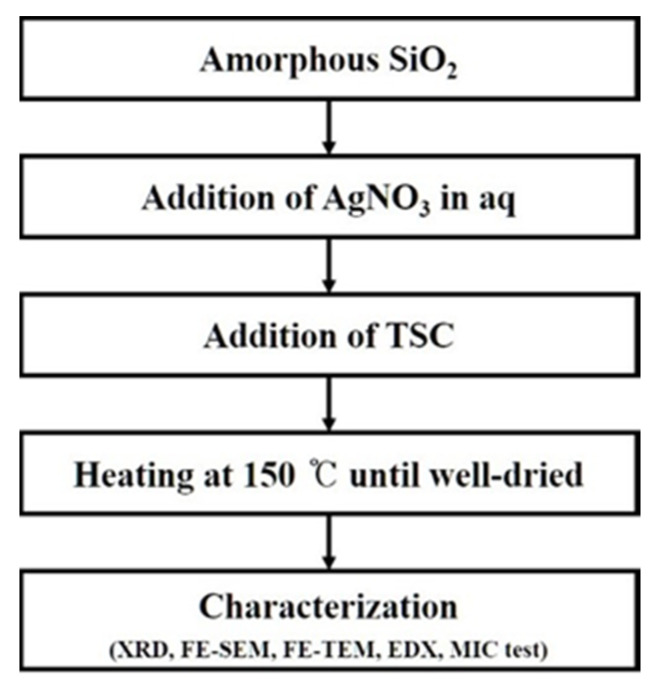
The synthesis procedure of SiO_2_@Ag mesoporous nanoparticles.

The SiO_2_@Ag mesoporous nanoparticles were synthesized by the impregnation method using TSC as a reducing agent. The previously prepared silica spheres without calcination (0.03 g) and AgNO_3_ in an aqueous medium (10 mL; 0.002 M) were mixed for 30 min on the magnetic stirrer. Then, TSC (1 mL; 0.02 M) was added dropwise to the previously prepared solution under continuous stirring. Then, the mixture was heated to 150 °C and maintained at this temperature until the solvent and volatile organic compounds were completely evaporated (Scheme 1).

### 2.4. Characterization of the SiO_2_@Ag Mesoporous Nanoparticles

The X-ray diffraction instrument (XRD, MiniFlex II, Rigaku Ltd., Tokyo, Japan) was used to analyze the structural properties of the prepared materials with the Cu Kα radiation (λ = 0.1546 nm). The phase identification was carried out using High Score Plus software. The microstructure study was performed on a field emission transmission electron microscopy (FE-TEM, JEM 2100F, Jeol Ltd., Tokyo, Japan) and field emission scanning electron microscopy (FE-SEM, CZ/MIRA I LMH, TESCAN Ltd., Kohoutovice, Czechia) instruments. The energy dispersive X-ray spectrometer (EDS genesis, Oxford Ltd., Oxford, UK) analysis was conducted during the FE-TEM measurement. The X-ray Photoelectron Spectroscopy (XPS, K-alpha Thermo Scientific Ltd., Waltham, MA, USA) measurement was performed to investigate the chemical state and composition of the obtained material. This analysis was conducted using a monochromatized Al Kα radiation. Binding energy was fitted to the C1s spectrum at the 284.6 eV from an extra source of carbon and deconvolution performed [40]. First, a gallium nitride (GaN) substrate (SiO_2_ substrate was not used to avoid peak overlapping) was cleaned with acetone, ethanol, and deionized water, then purged with N_2_ gas until it was fully dried. Then the distilled water-based colloidal solution sample containing the as-prepared powder was placed on the GaN substrate and dried on a hot plate to avoid damage induced by moisture to the instrument.

### 2.5. Measurement of Antibacterial Activity in an LB Liquid Medium

The antibacterial properties of the SiO_2_@Ag mesoporous nanoparticles were assessed against Gram-negative *Escherichia coli (E. coli)* and Gram-positive *Bacillus subtilis (B. subtilis)*. These bacteria were chosen to represent Gram-negative and Gram-positive types respectively because they were relatively common and well-known for antibacterial study. Typically, the LB medium in the test tubes was sterilized at 121 °C for 15 min and cooled to room temperature. Then, the bacterial stock solution (50 μL) was added to the LB medium. Hereupon, the different concentrations of the NP suspension (0.00, 0.10, 0.25, 0.50, 0.75, and 1.00 mg/mL) were added, and the mixtures were incubated at 28 °C for 12 h with a 140 rpm shaking rate. Bacterial growth was monitored by measuring the absorption at 660 nm every 4 h using a UV-vis spectrophotometer. Moreover, a negative control experiment was performed without the SiO_2_@Ag suspension. The morphological changes of the SiO_2_@Ag-treated bacteria were studied with FE-SEM. Moreover, untreated bacteria were investigated for comparison. The bacteria samples were prepared as indicated in our previous work [41].

### 2.6. Measurement of Antibacterial Activity on Agar Plates

The antibacterial activity of the SiO_2_@Ag-coated steel and polyethylene (PE) substrates was studied with *E. coli* and *B. subtilis* using the disk diffusion assay. The mixture of agar powder and LB medium was purified using an autoclave (121 °C for 15 min); then, the mixture was cooled to room temperature. The bacterial strains were streaked on the as-prepared agar plates. The raw SiO_2_@Ag particles were coated on the steel and PE substrates using inorganic glass water binders. The SiO_2_@Ag-coated substrates were put on the bacteria-injected agar plates and placed in an oven incubator for 24 h at 28 °C. Finally, the inhibition zones were measured with a ruler, which indicated the bactericidal activity of the coated material on both steel and PE substrates.

## 3. Results and Discussion

The Stöber process was used to prepare the monodispersed SiO_2_ spheres [42]. The SiO_2_ spheres were collected, centrifuged, washed, and dried. Then, the Ag NPs were attached to the silica surface by applying AgNO_3_ and TSC as the source of Ag^+^ ion and reducing agent, respectively. Various reducing agents can be used to reduce Ag^+^ ions to make Ag NPs [35,43,44]. Sodium borohydride and hydrazine hydrate are the most common reductants. However, the attachment of metal NPs on the template is difficult to achieve because of weak bonding strength. Hence, TSC as a reducing agent is employed to make Ag NPs and support strong interaction with the template. SiO_2_, as the template, was added to the AgNO_3_ aqueous solution before the redox reaction to ensure good dispersion and chemisorption of Ag^+^ ions on the negatively charged SiO_2_ surface. Then, TSC was added under a heated environment to reduce Ag^+^ ions producing the Ag NPs. Typically, it is difficult to reduce Ag^+^ ions with TSC without heat treatment. Thus, we applied heat to achieve reduction and nucleation. Here, we report an inexpensive one-pot synthesis method for SiO_2_@Ag with Ag NPs (5–15 nm size) on SiO_2_ particles. The synthesized NPs were used to destroy *E. coli* and *B. subtilis* bacteria.

**Figure 1:**
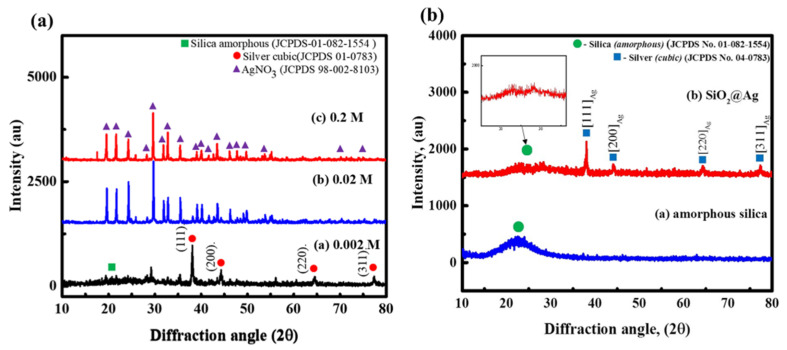
The XRD pattern of (**a**) different concentrations of AgNO_3_ for Ag NPs’ formation and (**b**) amorphous silica with SiO_2_@Ag patterns. The amplified image indicated by the arrow reveals an amorphous silica peak.

The XRD analysis was carried out to investigate the structure of amorphous SiO_2_ and SiO_2_@Ag, as shown in Figure 1. Pure amorphous SiO_2_ exhibits a peak with a broad diffraction angle of 14.78–33.75°, which corresponds to JCPDS No. 01-082-1554 in Figure 1a. The XRD pattern in Figure 1b exhibits four diffraction peaks at 37.98, 44.1, 64.38, and 77.32°, which correspond to the (111), (200), (220), and (311) crystal planes of cubic Ag NPs (JCPDS No. 04-0783). As shown in the magnified image (Figure 1, inset), the XRD pattern exhibits only an amorphous peak, which indicates the presence of amorphous silica.

**Figure 2:**
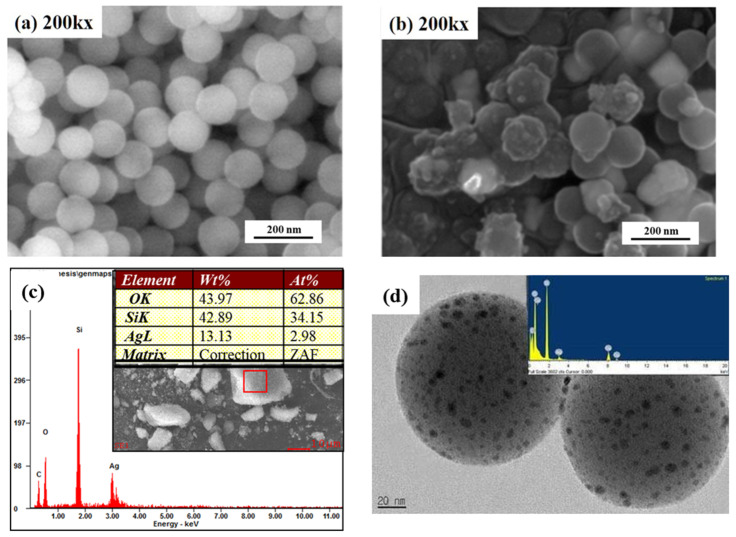
The FE-SEM images of (**a**) amorphous SiO_2_ and (**b**) SiO_2_@Ag; (**c**) EDX analysis of SiO_2_@Ag; (**d**) FE-TEM image of SiO_2_@Ag with EDX analysis (inset).

The FE-SEM images of the spherical silica (approximately 120 nm diameter) and SiO_2_@Ag are depicted in Figure 2a,b. As shown in Figure 2b, it is observed that both bare and Ag NP-coated SiO_2_ are presented which is consistent with the FE-TEM image. The SiO_2_@Ag mesoporous NPs were formed by reducing Ag^+^ ions on the silica support which is attributed to the electrostatic interaction between the negatively charged amorphous SiO_2_ and Ag^+^ ions [45]. The FE-TEM analysis was performed to confirm the deposition of Ag NPs on SiO_2_ (Figure 2c). Figure 2c shows the successful deposition of Ag NPs on the silica surface. The average size of Ag NPs was in the range of 5–15 nm (Figure 2d). The particle size indicates that the one-pot synthesis route allows the impregnation of metal NPs even at low concentrations of the metal precursor while maintaining its inherent properties. This result suggests that the synthesized NPs can maintain their antibacterial activity because when the particles become larger, their antibacterial activity declines [46]. The EDX analysis was performed to evaluate the silver and silica as indicators of the Ag NP attachment. This analysis is shown in the magnified image (Figure 2c,d, inset). The major emission energy peaks for silica and silver are observed in the range of 1.6–2.0 keV and 2.5–3.5 keV, respectively. This result confirms that Ag NPs have been attached to the SiO_2_ template.

**Figure 3:**
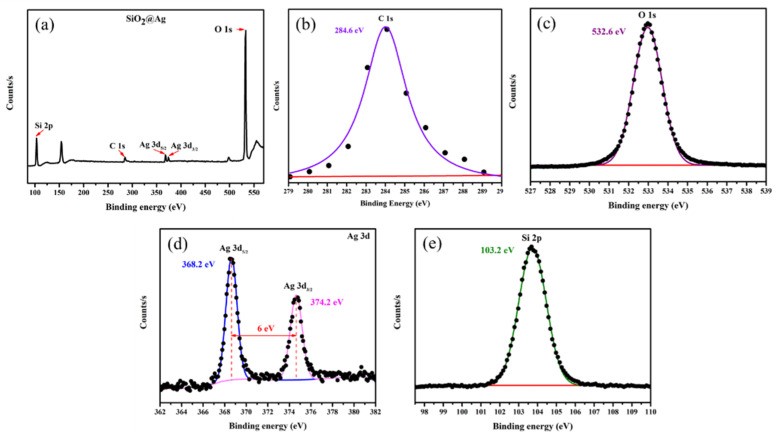
The XPS measurement of SiO_2_@Ag mesoporous NPs: (**a**) Survey spectrum; (**b**) C1s; (**c**) O 1s; (**d**) Ag 3d; (**e**) Si2p spectra.

The chemical state and the elemental composition of the SiO_2_@Ag structures are studied through an XPS analysis. Figure 3a shows the survey spectra of XPS of Ag NPs created on the SiO_2_ nanoparticles. The high-resolution spectra of C1s, O1s, Ag3d, and Si2p are depicted in Figure 3b–e, respectively. Figure 3b shows the symmetric C1s spectra at the 284.6 eV, which was fitted to the reference binding energy [32]. The substrate material, SiO_2_, was confirmed by the presence of Si2p peak at 103.2 eV, in agreement with the value of SiO_2_, shown in Figure 3e. Furthermore, the occurrence of O1s peak at 532.6 eV is attributed to the surface oxygen of SiO_2_ (Figure 3c). The two main peaks at 368.2 eV and 374.2 eV corresponds to the Ag 3d_5/2_ and Ag3d_3/2_ with the doublet at 6 eV indicates the existence of metallic silver (Ag^0^) on the substrate material [47], which is in good agreement with the XRD analysis (Figure 1b). Regarding the Stöber reaction, Santos et al. [48] obtained anticorrosive silica-based material by the sol-gel process applying TEOS. Silica was obtained through this process and Si2p binding energy was located at 103.5 eV. Moreover, this was confirmed by the existence of O1s peak at 532.8 eV which is related to the O-Si bonds. France et al. [49] also prepared core-shell magnetite@silica and the SiO_2_ layer was also obtained by the addition of TEOS precursor. Based on the XPS study, the Si2p peak has located at 103.5 eV which is also in good agreement with our SiO_2_ peak. They reported that the condensation reaction was followed by hydrolysis, which supports the loss of water and the creation of four oxygen bonds to the Si.

Therefore, if an incomplete hydrolysis reaction has occurred, several peaks would appear in the Si2p peak after deconvolution and those peaks will be related to the partially hydrolyzed TEOS sample. However, based on our Si2p peak shape, we believe a complete hydrolysis and condensation reaction has occurred because there is an absence of broadening or tails in the Si2p peak. Further, the condensation reaction is favorable to releasing OH molecules, which can be the reason for the complete formation of SiO_2_. This result can be confirmed by the O1s peak which is in good agreement with that of SiO_2_. Lastly, each peak value was slightly increased compared to the reference data. This also indicates that our synthesized SiO_2_ was completely condensed.

**Figure 4:**
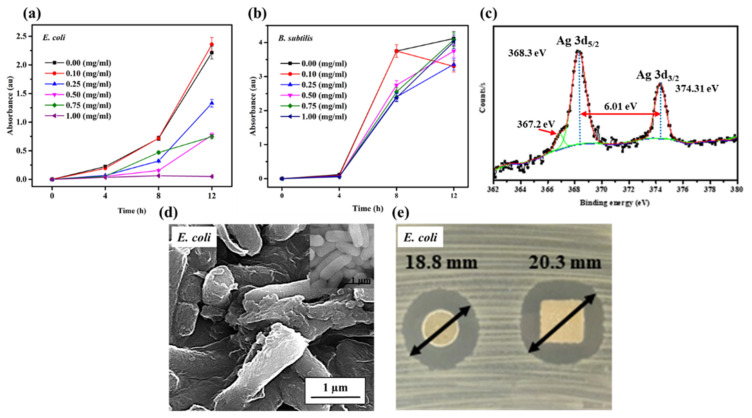
Investigation of the SiO_2_@Ag-based antibacterial agent: (**a**,**b**) Minimum inhibitory concentration (MIC) test against *E. coli* and *B. subtilis*; (**c**) XPS analysis of Ag NPs after treatment for MIC test; (**d**) FE-SEM image of the decayed morphology of the *E. coli* bacteria. The inset is the untreated *E. coli* used for the comparison. The scale bar is 1 μm; (**e**) Disk diffusion assay of SiO_2_@Ag coated on steel (round) and polyethylene (rectangular) versus *E. coli.*

The minimum inhibitory concentration (MIC) test was performed to estimate the antibacterial activity of the SiO_2_@Ag mesoporous spheres against *E. coli* and *B. subtilis* bacteria (Figure 4a). Notably, the SiO_2_@Ag NPs exhibited excellent dispersibility in aqueous media owing to the one-pot synthesis route and the use of TSC as a reducing agent. The antibacterial activity was determined at the SiO_2_@Ag concentrations of 0.00, 0.10, 0.25, 0.50, 0.75, and 1.00 mg/mL for 12 h with the time interval of 4 h. As shown in Figure 4a, the bacterial growth decreased with the increase of the NP concentration. When the concentration of the SiO_2_@Ag NPs was 1.00 mg/mL, the growth of the *E. coli* strain was fully restricted. This observation indicates that the MIC of the SiO_2_@Ag NPs for *E. coli* is 1 mg/mL. The *E. coli* bacteria has a negative charge on the cell wall that usually supports the electrostatic interaction between the Ag NPs and bacteria [50]. The released Ag+ ions penetrate the cell wall and interact with the thiol groups of the proteins which leads to the obstruction of the DNA replication followed by bacterial death [51]. However, as shown in Figure 4b, the SiO_2_@Ag mesoporous with NPs up to 1.00 mg/mL concentrations did not exhibit antibacterial activity against the *B. subtilis* strain which could be caused by the thick cell wall of Gram-positive bacteria [23]. Therefore, the prolonged release of silver ions in media could also be proven by partially oxidized Ag NPs after 24 h-long treatment shown in the additional XPS analysis (Figure 4c). D’Agostino et al. showed that anisotropic silver nanoplates on glass showed strong antibacterial activity, which is based on two different mechanisms: silver ion release and hyperthermia caused by the photo-thermal effect. They asserted that the silver release involves the formation of an Ag_2_O layer on the water-exposed surface and that slowly released Ag+ ions are replaced by Ag oxidized from the bulk [52]. The two major peaks of metallic Ag peaks are at 368.3 and 374.31 eV while a small part of the silver oxide (AgO) peak is at 367.3 eV. The major peak of Ag NPs had a doublet of 6.01 eV which corresponds with the existence of metallic silver (Ag0). These are the good evidence of constantly released Ag+ ion and stability of metallic silver in media, which supports the antibacterial activity [53]. Based on the MIC test results, an additional assessment of bacterial (*E. coli*) cell damage was performed using the FE-SEM analysis. The lowest concentration of the NPs (0.1 mg/mL) at which the bacterial growth was restricted was chosen as indicated in Figure 4d. The untreated *E. coli* (round shape, approximately 1.2-μm size) was used as the control sample. The bacterial cell wall of the SiO_2_@Ag-treated sample was wrinkled and partially damaged. This result shows that the bacterial growth was restricted at the lowest concentration even after 12 h and this observation correlates well to the MIC test data.

The practical application of the SiO_2_@Ag mesoporous NPs was studied using the disk diffusion assay against the *E. coli* strain. The SiO_2_@Ag mesoporous was coated on the steel and PE substrates. Importantly, the diameters of the inhibition zones (Figure 4e) were about 20.3 and 18.8 mm for *E. coli*, respectively. Thus, the SiO_2_@Ag mesoporous NPs are applicable as a coating material for both steel and plastic material surfaces.

## 4. Conclusions

In this report, we investigated a one-pot synthesis route for SiO_2_@Ag mesoporous spheres, which can be used as a highly effective antibacterial and anti-fouling agent. This synthesis route is also a compelling approach to impregnate metal nanoparticles on a template material. The obtained SiO_2_ template is formed into a spherical shape that ensures excellent attachment of metal ions and provides a high surface to volume ratio for metal NPs. XPS measurement was conducted to confirm O1s, Si2p, and Ag3d peaks which were in agreement with a value of SiO_2_ and Ag NPs. The prepared SiO_2_@Ag mesoporous spheres exhibited a more notable antibacterial activity against the E. coli strain compared with that of the *B. subtilis* bacteria which might be due to the thinner cell wall of Gram-negative type E. coli bacteria. Notably, SiO_2_@Ag mesoporous NPs exhibited an outstanding inhibition zone against E. coli bacteria as a coating material for both steel and polyethylene surfaces. In summary, the synthesized NPs were found to be an excellent coating material for both steel and polymer surfaces to restrict the growth of microorganisms.
